# Structural Characterization of the Mechanosensitive Channel Candidate MCA2 from *Arabidopsis thaliana*

**DOI:** 10.1371/journal.pone.0087724

**Published:** 2014-01-27

**Authors:** Hideki Shigematsu, Kazuko Iida, Masataka Nakano, Pratima Chaudhuri, Hidetoshi Iida, Kuniaki Nagayama

**Affiliations:** 1 Okazaki Institute for Integrative Bioscience, National Institutes of Natural Sciences, Okazaki, Aichi, Japan; 2 Biomembrane Laboratory, Tokyo Metropolitan Institute of Medical Science, Setagaya-ku, Tokyo, Japan; 3 Department of Biology, Tokyo Gakugei University, Koganei, Tokyo, Japan; 4 National Institute for Physiological Sciences, National Institutes of Natural Sciences, Okazaki, Aichi, Japan; 5 Department of Physiological Sciences, Graduate University for Advanced Studies, Okazaki, Aichi, Japan; University of South Florida College of Medicine, United States of America

## Abstract

Mechanosensing in plants is thought to be governed by sensory complexes containing a Ca^2+^-permeable, mechanosensitive channel. The plasma membrane protein MCA1 and its paralog MCA2 from *Arabidopsis thaliana* are involved in mechanical stress-induced Ca^2+^ influx and are thus considered as candidates for such channels or their regulators. Both MCA1 and MCA2 were functionally expressed in Sf9 cells using a baculovirus system in order to elucidate their molecular natures. Because of the abundance of protein in these cells, MCA2 was chosen for purification. Purified MCA2 in a detergent-solubilized state formed a tetramer, which was confirmed by chemical cross-linking. Single-particle analysis of cryo-electron microscope images was performed to depict the overall shape of the purified protein. The three-dimensional structure of MCA2 was reconstructed at a resolution of 26 Å from 5,500 particles and appears to comprise a small transmembrane region and large cytoplasmic region.

## Introduction

Plants respond to various environmental stimuli, including light, temperature, wind, and touch, all of which affect growth and development. Wind and touch are examples of mechanical stimuli that generate a Ca^2+^ signal in the cytoplasm [1]. This signal is thought to be generated through Ca^2+^-permeable, mechanosensitive (MS) channels in cellular membranes. Bacterial and animal MS channels have so far been studied extensively and characterized in detail at the molecular level [2]–[4]. In contrast, although the physiological roles of plant MS channels have long been implicated in thigmotropism and gravitropism, their molecular natures have just begun to be elucidated (reviewed in [5]–[8]),[9].

Homologues of the bacterial MS channel MscS, named MscS-like proteins or MSLs, have recently been identified and characterized in *Arabidopsis thaliana* [5], [10]–[15]. However, MS channels unique to eukaryotes, such as animal TRP channels, have not been found in plants. We previously identified genes that code for potential MS channels or their regulators in *Arabidopsis*, named *MCA1* and its paralog *MCA2* [16], [17]. MCA1 and MCA2 are composed of 421 and 416 amino acid residues, respectively, and share 73% full-length identity in amino acid sequence. *MCA1* cDNA was first isolated from an *Arabidopsis* cDNA library by functional complementation of a yeast *mid1* mutant defective in a putative Ca^2+^ channel component. MCA1 and MCA2 have been shown to mediate the influx of Ca^2+^, and MCA1-GFP and MCA2-GFP are located in the plasma membrane [16], [17]. Mechanical stress appears to activate MCA1. First, hypotonic shock increased [Ca^2+^]_cyt_ to a greater extent in *MCA1*-overexpressing (*MCA1*ox) seedlings than in control seedlings. Second, the accumulation of Ca^2+^ was approximately two-fold higher in the roots of *MCA1*ox plants than in those of wild-type roots. Third, MCA1 expressed in CHO cells increased [Ca^2+^]_cyt_ in response to membrane stretching. Fourth, the primary roots of *mca1*-null seedlings failed to penetrate the harder, lower agar medium from the softer, upper agar medium of a two-phase agar system, which indicated that MCA1 is responsible for touch sensing and/or signal transduction. Finally, MCA1 and MCA2 enhanced MS channel activity in the *Xenopus* oocyte plasma membrane [18]. MCA2 was shown to be involved in the uptake of Ca^2+^ in *Arabidopsis* roots [17]. These findings indicate that MCA1 and MCA2 play important roles in a Ca^2+^-permeable MS channel system.

In spite of these findings and the importance of MS channels in plant cells, the structures of the MCA proteins have yet to be elucidated. However, some progress has recently been made in determining their structure-function relationships [19]. The N-terminal region of both MCA1^1-173^ and MCA2^1-173^ was shown to be necessary and sufficient for Ca^2+^ influx when assayed in a yeast expression system. In relation to the present study, both MCA1^1-173^ and MCA2^1-173^ have unique predicted transmembrane (TM) segments near the N-terminus. Other structural information reported to date for the MCA proteins is that they form a dimer and tetramer, as revealed by non-reducing SDS-PAGE followed by Western blotting of yeast total extracts containing these proteins [19].

Recombinant expression and purification are important for determining MCA protein structures, but may be inappropriate for a recombinant expression system with cultured cells. Although the overexpression of MCA proteins induced cell death in *Escherichia coli* and mammalian cells, their constitutive expression in yeast cells and transient expression in CHO cells were successfully used for functional analyses [16], [17], [19]. However, MCA proteins have not been expressed in sufficient quantity for purification. This shortage appears to have been caused by the spontaneous influx of Ca^2+^ mediated by the MCA proteins. Therefore, in the present study, we employed a viral expression system in insect cells. The baculovirus system using Sf9 cells was shown to be useful for the expression and purification of eukaryotic proteins, including channel proteins, for structural analysis by electron microscopy [20], [21]. As mentioned above, the MCA2 protein is a small membrane protein that forms a tetramer in the membrane, the total mass of which may be approximately 200 kDa. Because this is considered to be a challengingly small particle for three dimensional (3D) reconstruction from cryo-electron microscopy (cryo-EM) images [22], [23], Zernike phase contrast (ZPC) cryo-EM was used in the present study to more clearly visualize small proteins in a detergent-solubilized state. Since ZPC cryo-EM has been applied to the typical cryo-EM applications [21], [23]–[29], it can be utilized to tackle difficulties in cryo-EM application due to the contrast issue of conventional, defocus phase contrast cryo-EM. ZPC cryo-EM has been used to successfully visualize small proteins including the Helicobacter pylori VacA toxin, an 88 kDa protein [30] and human Dicer, a 220 kDa asymmetric protein with its substrate RNA molecules [23].

In the present study, we established recombinant expression using the baculovirus expression system and purification of the MCA2 protein in a detergent-solubilized state. The purified protein was identified as a tetramer from chemical cross-linking and its overall shape was depicted by single-particle analysis with cryo-EM. The 3D structure reconstructed here suggests that MCA2 has a small TM region with a relatively large cytoplasmic region.

## Materials and Methods

### Construction of Baculovirus Carrying MCA1 and MCA2 and Infection of Sf9 Cells

The baculovirus containing *Arabidopsis MCA1* or *MCA2* cDNA was constructed by using the Bac-to-Bac Baculovirus Expression System (Invitrogen Japan KK, Tokyo, Japan). *MCA1* or *MCA2* cDNA having *Bam*HI and *Sal*I sites just upstream of the initiation codon and stop codon, respectively, was synthesized by PCR. The PCR products were cut with *Bam*HI and *Sal*I and inserted between the *Bam*HI and *Sal*I sites of the YEplac112-based multicopy expression vector YEpTDH-6HC [*TRP1*], in which an inserted cDNA was transcribed with a 6xHis tag sequence at the 3′-end under the control of the *TDH3* promoter of the yeast *Saccharomyces cerevisiae*. The resulting plasmids were designated YEpT-MCA1-6H and YEpT-MCA2-6H, respectively.

The *Bam*HI-*Not*I fragments of the plasmids described above, *i.e*. YEpT-MCA1-6H and YEpT-MCA2-6H, were inserted between the *Bam*HI and *Not*I sites of pFastBac1 (Invitrogen Japan KK). The resulting plasmids were introduced into the *E. coli* strain DH10Bac (Invitrogen Japan KK) to isolate Bacmid DNA carrying MCA1-6H or MCA2-6H. Bacmid DNA was purified and used to produce the baculovirus with Sf9 cells according to the procedure described by the manufacturer of the Bac-to-Bac expression system (Invitrogen Japan KK). The resulting baculovirus was amplified five times to obtain a high-titer virus stock and was then used for protein expression. We examined the expression profiles of MCA1-6H and MCA2-6H by varying the multiplicity of infection (MOI) and time post-infection. The amount of recombinant protein expressed was visualized by Western blotting using an anti-His-Tag polyclonal antibody as a primary antibody (MBL Co., Ltd., Nagoya, Japan), anti-MCA1 polyclonal antibody (designated Apep1 IIDA1), or anti-MCA2 polyclonal antibody (designated Bpep4) and a secondary antibody conjugated with alkaline phosphatase. SDS-PAGE was performed using NuPAGE 4–12% BisTris Gel and the MES buffer System (Invitrogen Japan KK).

### Ca^2+^ Accumulation in Yeast Cells

Strain H311 of the yeast *Saccharomyces cerevisiae* (*MAT* **a** *mid1-*Δ5::*HIS3 his3*-Δ*1 leu2-3*,*112 trp1-289 ura3-52 sst1-2*), which is defective in Ca^2+^ uptake [31], was transformed with a plasmid, such as YEpT-MCA1-6H, YEpT-MCA2-6H, or YEpTDHXho (empty vector). The transformed cells were grown to the exponentially growing phase (approx. 2×10^6^ cells/ml; 1 ml) in SD.Ca100 medium at 30°C and subjected to Ca^2+^ accumulation assays as described by Iida *et al.* [32]. The cells were incubated for 0 and 2 h at 30°C with 185 kBq/ml ^45^CaCl_2_ (1.81 kBq/nmol; PerkinElmer, Boston, MA; Cat. no. NEZ013) and an aliquot (100 µl; duplicate) was taken, filtered on a Millipore filter (type HA; 0.45 µm) presoaked in 5 mM CaCl_2_, and washed five times with 5 ml of the same solution. The radioactivity retained on the filter was counted with the scintillation cocktail ReadyProtein (Beckman Coulter K.K., Tokyo, Japan) in a liquid scintillation counter (Beckman Coulter K.K.).

### Ca^2+^ Accumulation in Insect Sf9 Cells

Sf9 cells infected with a recombinant baculovirus containing MCA1-6H, MCA2-6H, or β-glucuronidase (control) at an MOI of 5.0 were assayed for activity to accumulate Ca^2+^. The infected cells were grown for 48 h in serum-free medium (SF-900II SFM, Invitrogen). The cells (approx. 6×10^6^ cells) were then collected by centrifugation for 3 min at 1,500 rpm and 25°C. The pellet was washed once with wash buffer (154 mM NaCl and 10 mM MOPS, pH 7.4) by centrifugation as above and resuspended in 6 ml of uptake solution (25 µM CaCl_2_, 154 mM NaCl, and 10 mM MOPS, pH 7.4). Part (0.5 ml; duplicate) of the suspension was used for Bradford assays to determine protein contents, and the remainder was incubated for 0 and 30 min with 11.1 kBq/ml ^45^CaCl_2_ (0.444 kBq/nmol). An aliquot (0.5 ml; duplicate) was removed, filtered on a Millipore filter (type HA; 0.45 µm) presoaked in filtration solution (154 mM NaCl, 1 mM EGTA), and washed five times with 5 ml of the same solution. The radioactivity retained on the filter was measured as described above.

### Protein Purification

Sf9 cells were grown to a density of 2.0×10^6^ cells/ml with a suspension culture at 27°C in serum-free medium and then infected with the baculovirus containing MCA2-6H at a MOI of 5.0. After 48-h cultivation, cells were collected by centrifugation for 20 min at 4,000 g and 4°C. The cells were washed once with PBS (150 mM NaCl and 25 mM sodium phosphate buffer, pH 7.4), centrifuged as above, immediately frozen, and stored at −30°C until used. The frozen cells were suspended in 10 volumes (v/w) of PBS containing a protease inhibitor cocktail (Complete EDTA-free protease inhibitor cocktail; Roche Diagnostics K.K, Tokyo) with a Teflon homogenizer. The cells were further disrupted by French Press (Thermo Fisher Scientific K.K., Yokohama) at a pressure of 10,000 psi. The cell lysate was centrifuged for 20 min at 4,000 g and 4°C to remove cell debris and the supernatant was then ultracentrifuged for 60 min at 100,000 g and 4°C. The precipitate was stored as the membrane fraction at −30°C.

The membrane fraction was homogenized with a Teflon homogenizer in 5 ml of PBS containing 8% ammonium perfluorooctanoate (APFO) (Wako Pure Chemical Industries, Ltd., Osaka) and the protease inhibitor cocktail described above. After incubation for 60 min at room temperature, the sample was diluted two-fold with PBS to reduce the concentration of APFO to 4%. After ultracentrifugation for 30 min at 10,000 g and 20°C, the APFO-soluble fraction was applied to a His GraviTrap column (GE Healthcare Japan K.K., Tokyo) equilibrated with PBS containing 4% APFO in advance. The column was washed with 20 ml of wash buffer (PBS containing 4% APFO), and the bound proteins were eluted with elution buffer (PBS containing 4% APFO, pH 6.0). The elution was neutralized with PBS containing 4% APFO (pH 8.0) and concentrated 10 times with the YM-100 Microcon centrifugal filter device (Millipore Japan, Tokyo). The concentrate was filtered with Ultrafree-MC Centrifugal Filter Units (0.22 µm) (Millipore Japan) and then purified by Superdex 200 (3.2/30) size exclusion chromatography in a SMART system (GE Healthcare Japan) with PBS containing 4% APFO at a flow rate of 40 µl/min. The elution of proteins was monitored by measuring absorbance at 280 nm and fractionated into 20-µl fractions. Protein concentrations were determined spectrophotometrically at 280 nm using an extinction coefficient as previously reported [33].

### Stoichiometric Analysis of Purified Protein

Glutaraldehyde was added to a final concentration of 10 mM, and incubated at room temperature up to 30 min for chemical cross-linking. A portion of the reaction mixture was used and the reaction has terminated by the addition of Tris-HCl (pH 8.0) to a final concentration of 50 mM. After a 15-min incubation, samples were subjected to SDS-PAGE with the NuPAGE 4–12% BisTris Gel and MOPS buffer system and visualized by Coomassie Brilliant Blue (CBB) staining. The purified protein was incubated with the SDS-PAGE sample buffer without dithiothreitol for SDS-PAGE under non-reducing conditions. Samples were then subjected to SDS-PAGE with the NuPAGE 4–12% BisTris Gel and MES buffer system and visualized by CBB staining.

### Cryo-electron Microscopy and Image Processing

Purified MCA2-6H was embedded in a thin layer of vitreous ice on a thin carbon film covered on a holey carbon grid. Commercially available holey carbon grids (R1.2/1.3 grids, Quantifoil MicroTools GmbH, Jena, Germany) were covered with an amorphous carbon film (about 5-nm thickness), dried in air, and kept in a desiccator in advance. The carbon film-covered Quantifoils were hydrated by glow-discharge at low air pressure just before the preparation of specimens. A total of 2.5 µl of MCA2-6H (0.05 mg/ml) was then applied, the excess solution was blotted with filter paper at 20°C with 100% humidity, and the grid was quickly placed into liquid ethane cooled by liquid nitrogen using Vitrobot Mark IV (FEI Company Eindhoven, the Netherlands). The grid was transferred into a cryo-EM, JEM-3100FFC (JEOL Co., Ltd, Tokyo, Japan) equipped with a field emission gun operated at an acceleration of 300 kV voltage, a Zernike phase plate, and an in-column (omega-type) energy filter. The temperature of specimens was maintained at ∼55 K as reported [30]. The Zernike phase plate was made of a vacuum-evaporated amorphous carbon film ∼27 nm thick which was optimized for 300 kV electrons [34]. The hole in the center of the film was 0.7 µm in diameter. The phase plate was kept at a temperature above 150°C to prevent beam-induced contamination [35]. Cryo-EM observations were performed with the Zernike phase plate inserted at the back focal plane of an objective lens to obtain in-focus Zernike phase contrast (ZPC) images. Data was collected at a nominal magnification of 60,000 in a zero-loss energy filtering mode with a 20 eV slit width at an electron dose of 20 e^-^/Å^2^. Images were recorded using the 2k×2k CCD camera, Megascan-795 (Gatan, Pleasanton, CA) with a pixel size of 0.3 nm.

Image processing and 3D reconstruction were performed using the EMAN version 1.9 [36], IMAGIC [37] and SPIDER [38] software packages. A total of 8,265 particles from CCD images were selected manually and boxed using the BOXER program from the EMAN package. Particles were then centered, low-pass filtered at 6 Å, and high-pass filtered for two Fourier pixels for initial image processing. A reference-free two-dimensional (2D) classification was performed with 8,265 particles into 200 classes by refine2d.py in the EMAN software package to visualize size variations in particles picked by semi-automated particle picking using the BOXER. A 3D model was generated using the startcsym command by applying C4 symmetry, which was suggested by the chemical cross-linking analysis. The initial model for projection matching refinement was made from this model by applying a low-pass filter to 70 Å and threshold masking using SPIDER. All micrographs were then filtered using Matlab script to suppress low-frequency signals as described elsewhere [23]. Particles were re-boxed from filtered micrographs with a box-size of 80×80 pixels. A reference-free 2D classification was performed for these particles using IMAGIC. Multivariate statistical analysis and multi-reference alignment were performed for 50, 200, 200, and 50 classes. Projection matching refinement was performed with the refine command in the EMAN package, applying C4 symmetry. Particles were classified into 133 classes corresponding to a 6-degree angular step during the iterative refinement procedure. The final 3D map was reconstructed after eight iterations. The resolution of the final map was estimated by the standard 0.5 criterion of the Fourier shell correlation between two maps made separately from two halves of the data set. The map was low-pass filtered to 20 Å and rendered into surface views with CHIMERA [39].

## Results

### Functional Expression of Tagged MCA1 and MCA2

**Figure 1:**
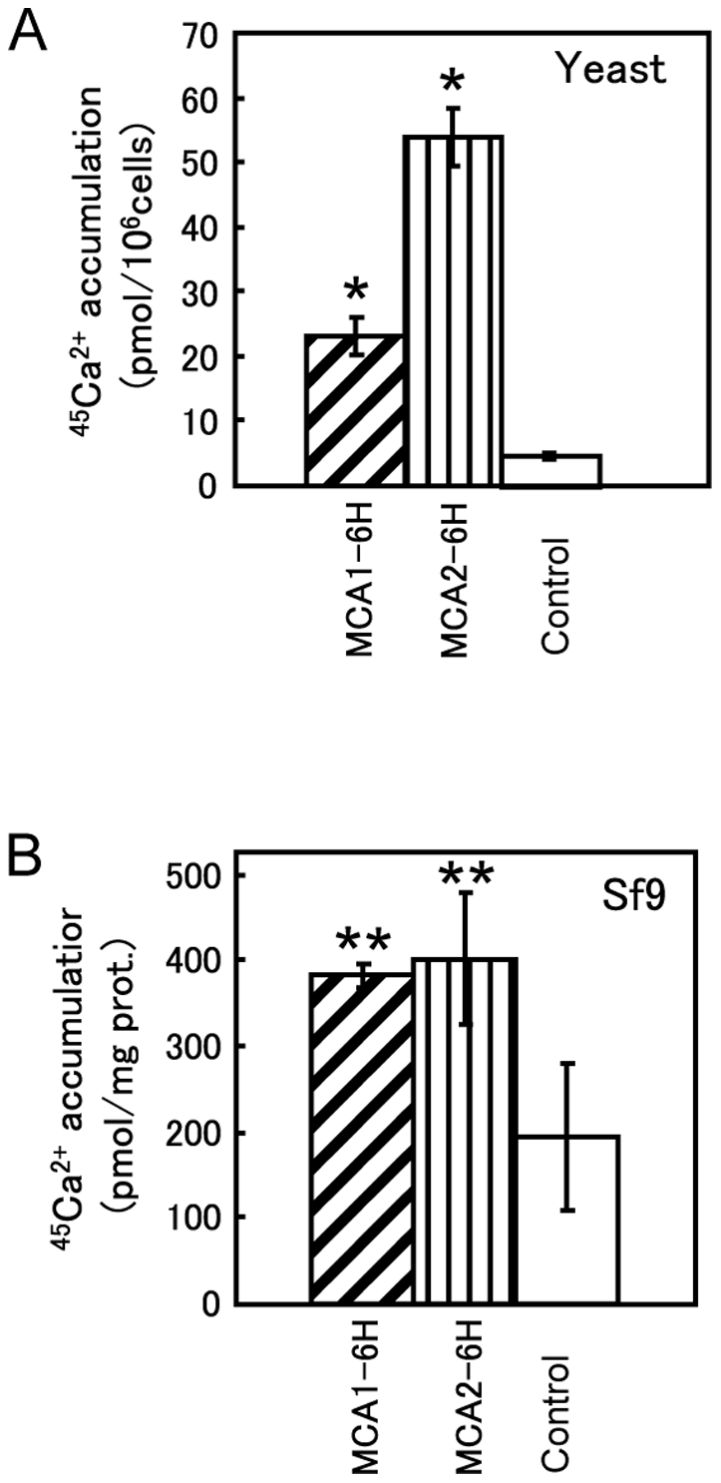
Carboxyl-terminally tagged MCA1 and MCA2 retained their activities. (**A**) Ca^2+^ accumulation in yeast *mid1* mutant cells carrying various plasmids. Cells were grown to the exponential phase in SD.Ca100 medium and incubated for an additional 2 h at 30°C with 185 kBq/ml of ^45^CaCl_2_ (1.81 kBq/nmol). The radioactivity that accumulated in these cells was measured as described in the Materials and Methods. The mean for at least three independent experiments (± SD) is shown for each yeast strain. The plasmids carried by the *mid1* mutant are as follows: YEpT-MCA1-6H (for MCA1-6H), YEpT-MCA2-6H (MCA2-6H), and YEpTDHXho (control). *, *p*<0.001 versus control. (**B**) Ca^2+^ accumulation in insect Sf9 cells expressing MCA1-6H or MCA2-6H. Cells were infected with a recombinant baculovirus carrying MCA1-6H or MCA2-6H cDNA at a MOI of 1.0 and incubated for 2 days at 25°C. Infected cells were harvested, washed, and resuspended in uptake solution as described in the Materials and Methods. The suspension was incubated for 30 min with 11.1 kBq/ml of ^45^CaCl_2_ (0.444 kBq/nmol). The radioactivity that accumulated in the cells was measured as described above. The mean for three independent experiments (± SD) is shown for Sf9 cells expressing MCA1-6H, MCA2-6H, or β-glucuronidase (control). **, *p*<0.05 versus control.

To purify MCA1 and MCA2, a 6xHis tag was fused to the carboxyl terminus of both proteins. Ca^2+^ accumulation activity was assessed in yeast *mid1* mutant cells expressing each of the proteins to determine if the resulting proteins, named MCA1-6H and MCA2-6H, retained their activities. Ca^2+^ accumulation is known to be defective in the *mid1* mutant [32] and, therefore, provides a good assay system for evaluating the Ca^2+^ accumulation activity of proteins of interest derived from other organisms [16], [17], [19], [40]. The accumulation of Ca^2+^was markedly higher in yeast cells expressing MCA1-6H or MCA2-6H than in control cells (Figure 1A). This result was quantitatively similar to our previous findings obtained with cells expressing non-tagged MCA1 and MCA2 [16], [17]. The activity of MCA2-6H was 2-fold higher than that of MCA1-6H.

**Figure 2:**
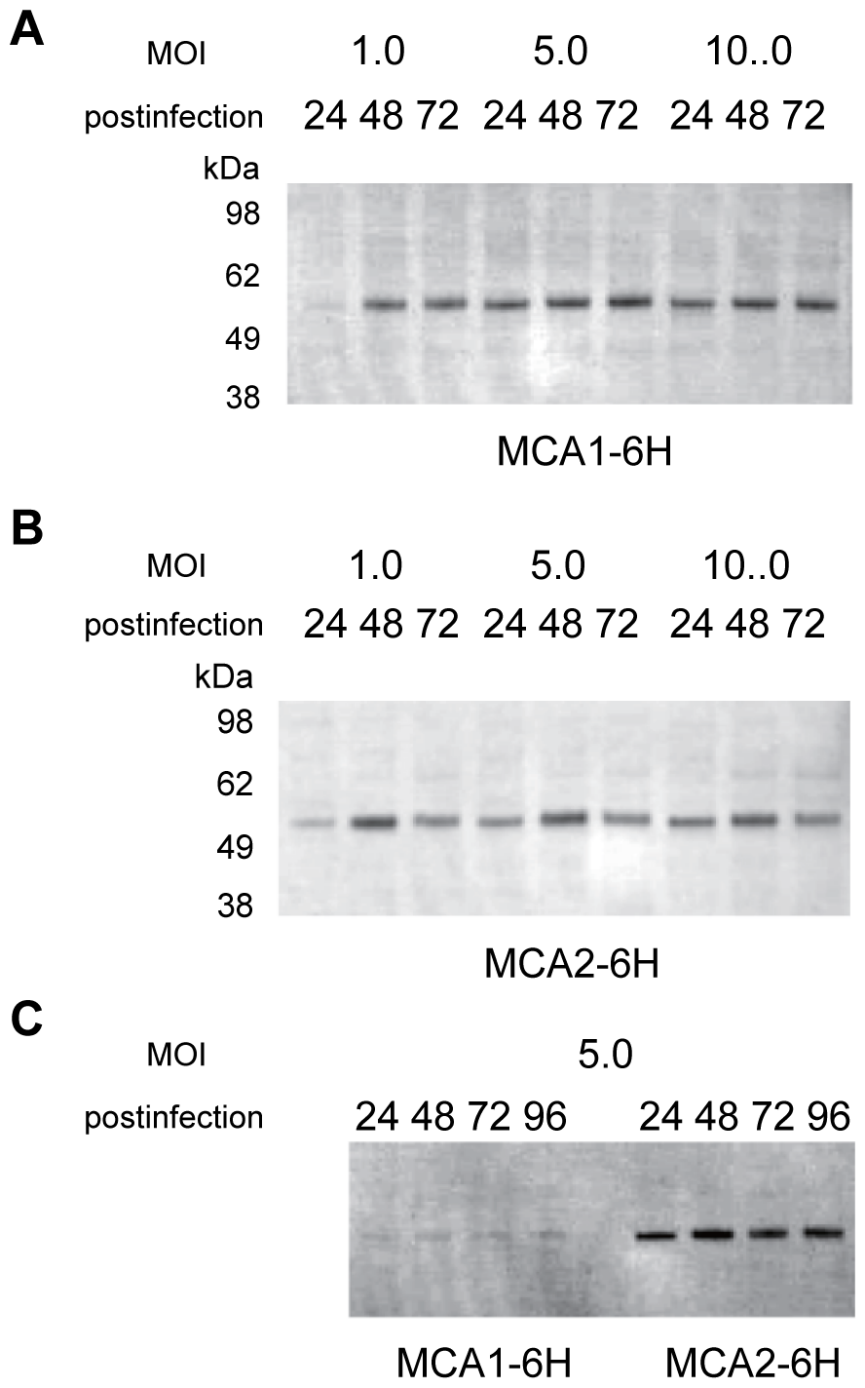
Expression profiles in Sf9 cells infected with a recombinant baculovirus. Western blot analysis of the expression of the MCA1-6H (**A**, **C**) and MCA2-6H (**B**, **C**) proteins using anti-MCA1 (**A**), anti-MCA2 (**B**), and anti-6xHis tag (**C**) antibodies. Differences in the post-infection times and MOI are shown above the panels.

We then examined the activities of MCA1-6H and MCA2-6H in insect Sf9 cells because we planned to isolate these proteins from Sf9 cells infected with a baculovirus containing either MCA1-6H or MCA2-6H. Pilot experiments to determine appropriate conditions for infection and cultivation revealed that the amount of both proteins did not increase after 48-h post-infection at the MOIs we examined (Figure 2A and B) and no obvious cell growth was observed after infection (data not shown). Thus, we routinely used 48-h post-infection cells at a MOI of 5.0. Ca^2+^ accumulation activity was approximately two-fold higher in Sf9 cells expressing MCA1-6H or MCA2-6H than in control Sf9 cells expressing β-glucuronidase (Figure 1B). Figure 2C shows the results of Western blotting using anti-His tag antibody as a primary antibody to detect the relative levels of MCA1-6H and MCA2-6H, which were clearly different from those shown in Figure 2A and B, in which antibodies specific to MCA1 or MCA2 were used. This result revealed a marked difference in the amount of protein expressed or how the attached 6xHis tag was exposed as an epitope. Therefore, MCA2-6H was selected instead of MCA1-6H because of the greater amount or accessibility of the 6xHis tag of MCA2-6H, which is advantageous for protein purification.

### Purification of MCA2-6H

**Figure 3:**
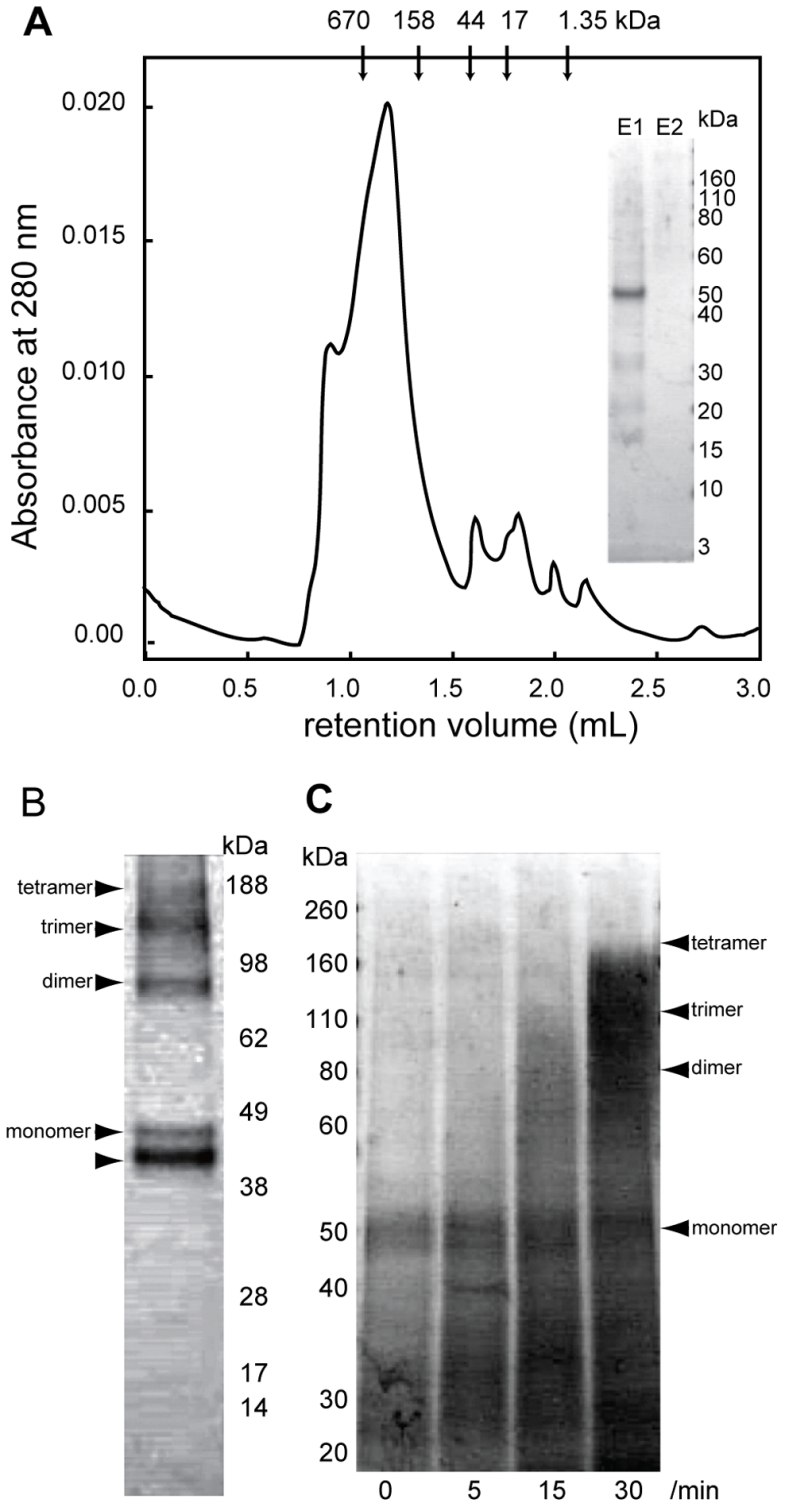
Size-exclusion chromatography and stoichiometry analysis. (**A**) Size-exclusion chromatogram of affinity-purified MCA2-6H. The retention volumes of each molecular weight standard are shown with molecular weights above the panel. The inset shows the results of SDS-PAGE visualized by CBB staining of the first and second elution (E1 and E2) from the affinity column. (**B**) SDS-PAGE under non-reducing conditions of purified MCA2-6H followed by CBB staining. Arrowheads indicate the possible monomer, dimer, trimer, and tetramer of MCA2-6H. The bottom arrowhead shows an additional band, which may correspond to a monomer with an internal disulfide bond of MCA2-6H. (**C**) SDS-PAGE under reducing conditions of cross-linked MCA2-6H followed by CBB staining. Arrowheads represent the positions of the monomer, dimer, trimer, and tetramer of MCA2-6H. The time (min) of crosslinking is shown under the bottom of each lane.

MCA2-6H was purified by affinity chromatography and size-exclusion chromatography. The detergent APFO used here has been employed to analyze the oligomeric structure of membrane proteins by electrophoresis and also to purify membrane proteins in their oligomeric state [41]–[43]. APFO-solubilized MCA2-6H appeared as a concentrated major band on CBB staining of the SDS-PAGE gel after affinity purification (inset in Figure 3A). Fraction 1 was further purified by size-exclusion chromatography. The chromatogram (Figure 3A) revealed that the majority of proteins appeared in a much higher molecular weight range than that of the MCA2-6H monomer. Some of the proteins appeared in the void volume, which indicated that MCA2-6H formed aggregates in solution. The chromatogram also showed one major peak between the molecular weight standards of thyroglobulin (670 kDa) and γ-globulin (158 kDa). Since detergent-solubilized proteins are often decorated with detergent molecules, it is plausible that the major peak appeared at a retention volume corresponding to a higher molecular weight than the actual molecular weight. Alternatively, the apparently high molecular weight of MCA2-6H may have been due to an oligomeric form of this protein in the APFO-solubilized state. We collected 20-µl fractions and used only the peak fraction for further analysis.

We performed SDS-PAGE analysis of the purified protein under non-reducing conditions (Figure 3B) and chemical cross-linking analysis of the purified protein followed by SDS-PAGE analysis under reducing conditions to reveal the stoichiometry of the oligomer (Figure 3C). Figure 3B shows four bands (indicated by arrowheads) whose molecular sizes corresponded roughly to a monomer, dimer, trimer, and tetramer and another band (indicated by an arrowhead in the bottom) just below the monomer, which appeared to be a monomer with an internal disulfide bond. On the other hand, chemical cross-linking analysis showed smeared out bands as a result of the migration of MCA2-6H to higher molecular weight ranges, and this was dependent on the reaction time of cross-linking (Figure 3C). A fraction of the bands migrated to the top, which was equivalent to the tetramer of MCA2-6H. following incubation for 30 min. These results indicate that MCA2-6H in an APFO-solubilized state is present as a tetramer.

### Cryo-EM and 3D Reconstruction

**Figure 4:**
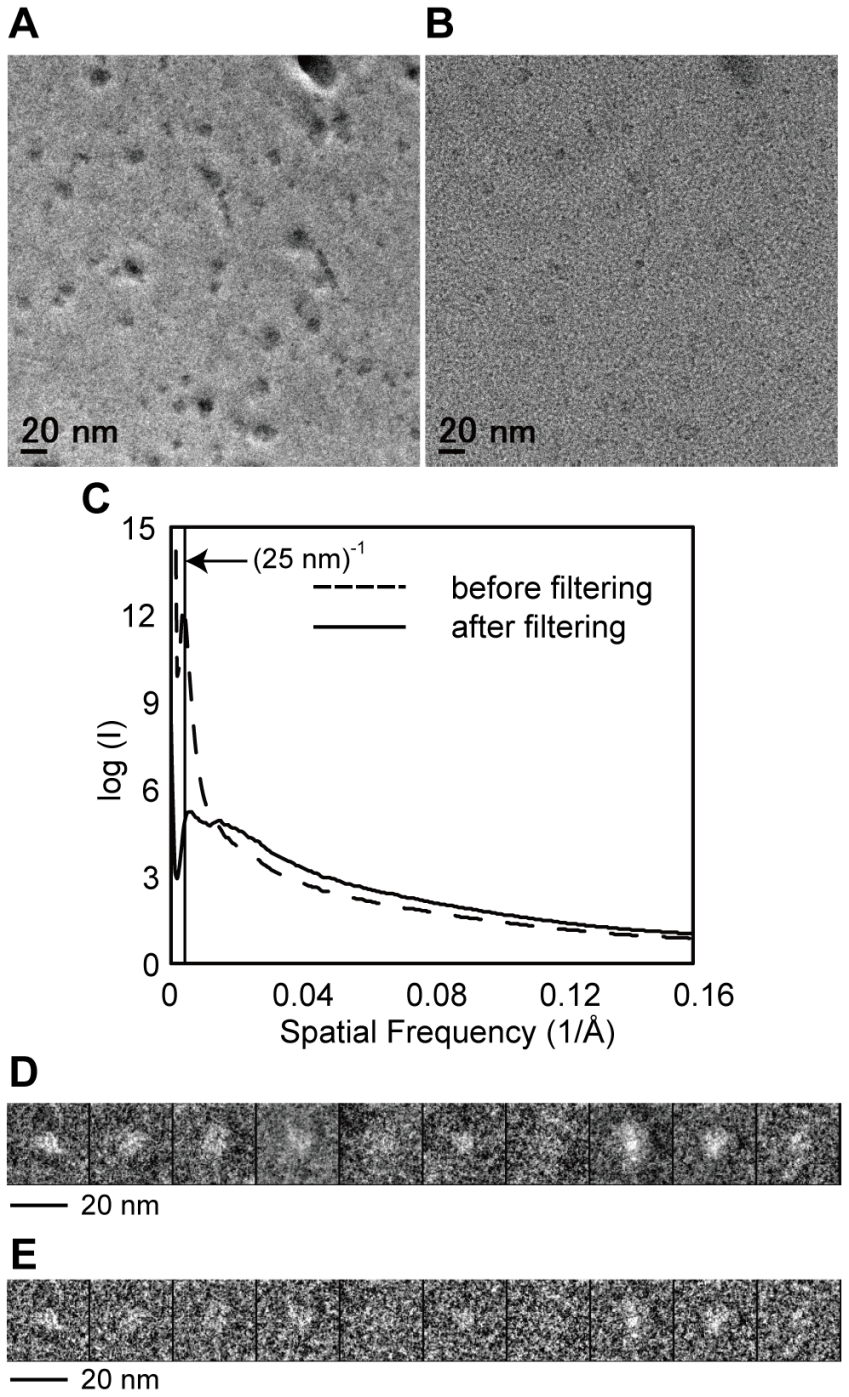
Cryo-EM micrographs of (A) ZPC cryo-EM and (B) defocus phase contrast cryo-EM. Those two images were taken at the same area under (**A**) in-focus and (**B**) 5 µm underfocused conditions. (**C**) Comparison of the rotationally averaged power spectrum of the ZPC cryo-EM micrograph before and after filtering. The dashed line represents an original ZPC cryo-EM image and the continuous line represents a filtered ZPC cryo-EM image. (**D**) (**E**) Examples of particles picked from original ZPC cryo-EM images and filtered ZPC cryo-EM images, respectively. The contrast of each particle stack has been inverted.

**Figure 5:**
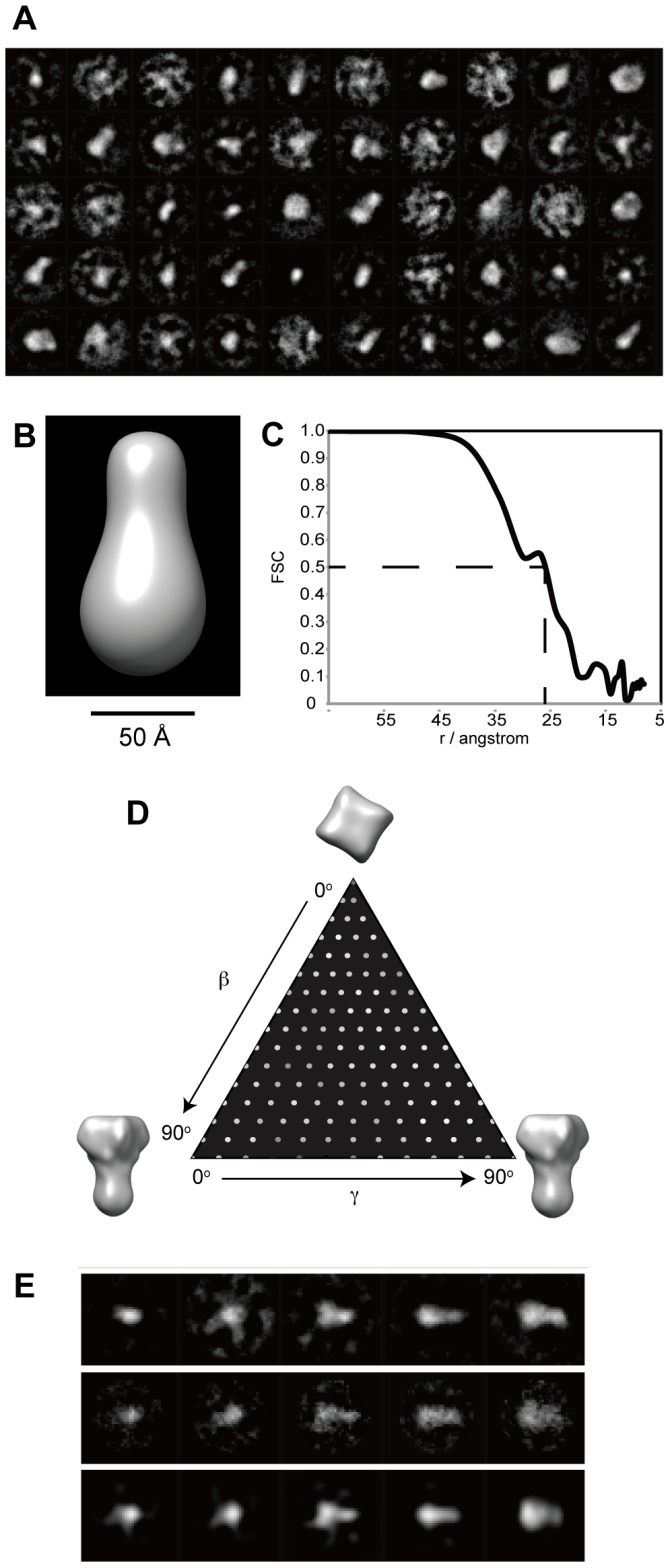
Cryo-EM structure reconstruction (A) Class-averages from the reference-free two-dimensional classification. Of the 200 total classes, 14 are shown. (**B**) Surface representation of the initial model that was used for projection-matching refinement. (**C**) Fourier shell correlation (FSC) between reconstructions from even and odd halves of the data set, plotted against spatial frequency. The value fell to the criterion level of 0.5 at a resolution of 26 Å. (**D**) Projection angle distribution. Each particle image represents a projection of the three-dimensional channel particle, with the projection direction defined by the Euler angles β and γ. The angle γ represents a rotation about the symmetry axis, and β is a rotation normal to the axis. Because of the four-fold symmetry, unique projection directions are described by values of both β and γ in the range 0 to 90°. The intensities of the dots in the figure represents the number of particle images assigned to each pair of β and γ values, and shows that the angle distribution was well sampled as required for good 3D reconstructions. (**E**) Comparison between reference-free 2D classification (top raw), classes for final reconstruction (middle raw), and reprojections of 3D reconstruction (bottom raw).

**Figure 6:**
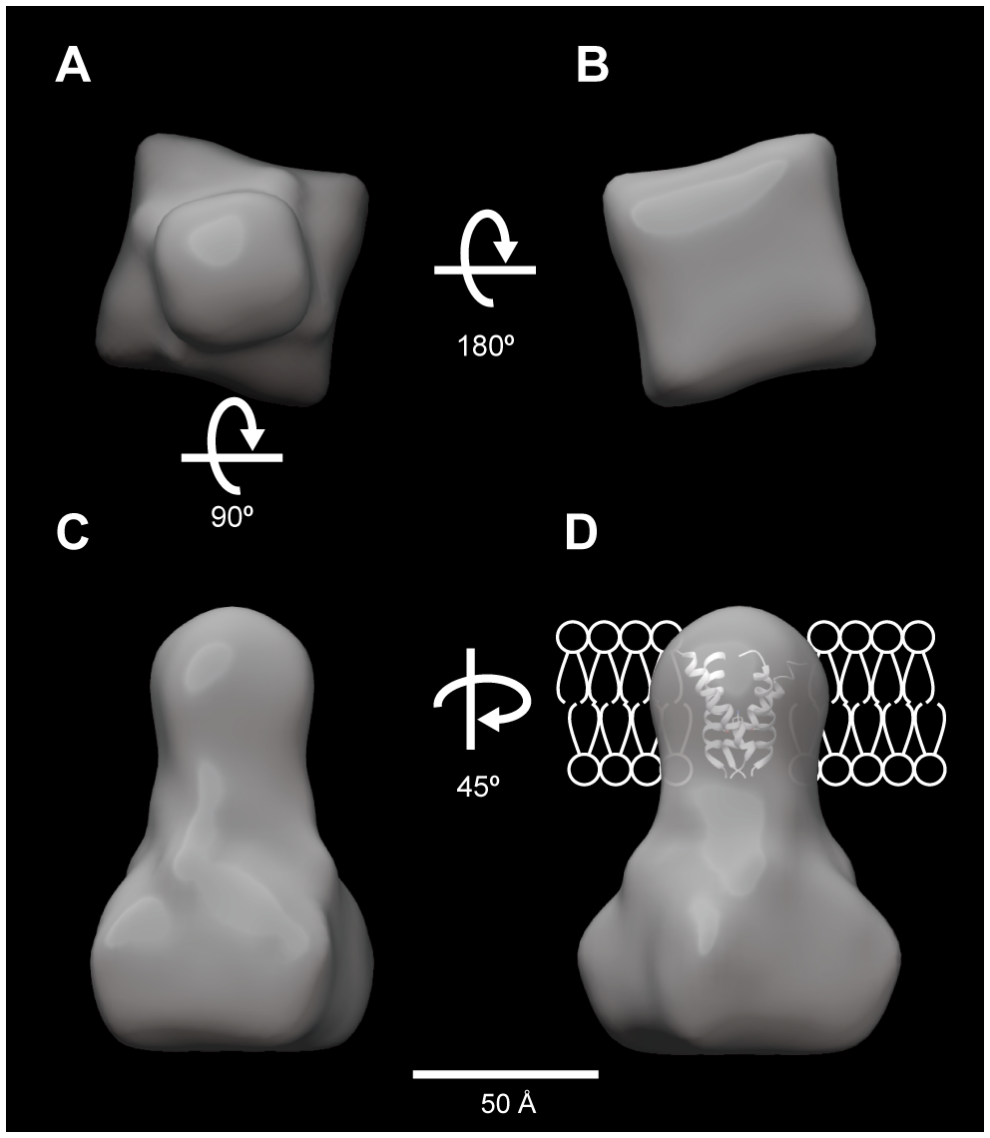
Surface representation of MCA2-6H viewed from four different angles. Surface representations of MCA2-6H corresponding to a molecular size of 200 kDa. (**A**) Top view, (**B**) bottom view, (**C**) side view, and (**D**) side view turned 45° in the z-axis, which is superposed with the crystal structure of the transmembrane segment of the M2 proton channel protein from influenza A virus (PDB #3C9J) and schematic illustration of the bilayer lipid membrane to propose membrane topology.

There are some successful results in negative stain of membrane proteins to visualize their molecular shape [44]–[46]. We used both a negative stain and cryo-EM to visualize MCA2-6H. Negative staining did not work well because of stain artifacts, which were attributed to the combination of the detergent and staining reagent (data not shown). We also had a practical issue regarding the variety of particle sizes even in cryo-specimens; however, particles of 5 to 15 nm in diameter were considered to be a reasonable size for a 200 kDa protein (Figure 4). Figure 4A and B show a comparison between ZPC and defocus phase contrast images (approximately 5 µm underfocused) of the same area. We had to choose particles in a relatively wide range (5 to 15 nm in diameter) because no structural information is available for MCA2ZPC. ZPC cryo-EM visualized particles with higher contrast of ice-embedded small proteins than defocus phase contrast images due to the conversion of contrast transfer function (CTF) from a sine to cosine function of spatial frequency [30], which made the selection of particles from cryo-EM images easier. The images taken here had a strong signal above a spatial frequency of (25 nm)^−1^ due to the physical size of the phase plate hole (Figure 4C). ZPC also revealed strong low frequency noises because a thin carbon film was used as a supporting film underneath the particles. We used a simple Matlab script instead of a Gaussian high pass filter for further image processing to compensate for the difference in the signal intensity between low and high frequencies. Figure 4C shows rotationally averaged power spectrums of a ZPC cryo-EM image before and after filtering. The low frequency intensity, which contained signals from particles and also noise from the supporting film, was dampened to avoid the alignment of particles being dominated by low frequency information. The filtered particles had less, but still sufficient contrast for further image processing (Figure 4D and E). The results obtained by reference-free 2D classification (Figure 5A) indicated that there are many particles that are too large or too small compared to the randomly generated 3D model with C4 symmetry (Figure 5B). We then used a relatively high bias (classkeep  = 0.2 for the refine command in EMAN package) not to keep particles in low scores in cross-correlation during the iterative refinement procedure and finally obtained a 3D model of MCA2-6H at 26-Å resolution from 5700 particles (Figure 5C). Euler angular distribution over the asymmetric unit used in the final reconstruction is shown in Figure 5D. The angles β and γ define the symmetry axis, whereas γ defines rotation about the symmetry axis. The brightness of the dots represents the number of particles assigned to the class, which is from 9 to 74 from dark to bright. Unfortunately, only a small number of particles were assigned to the top view, which is at the top of the triangle of the Euler angular distribution; therefore, we could not obtain a class-average in the reference-free two-dimensional classification, which is expected to show four-fold symmetry. Figure 5E shows comparisons between reference-free 2D class averages, classes for reconstruction, and reprojections of our 3D model. The gallery of reprojections was calculated from our 3D model and used for the comparison with reference-free 2D class averages. After the comparison, the classes for the final reconstruction corresponding to the matched reprojections were selected and displayed together with matched pairs in Figure 5E. Figure 6 shows the surface representation from four different angles. This model (EMDB-2313) consisted of two components with windmill cross-sections twisted 16° to each other. The height of the model was approximately 120 Å. The small part had dimensions of 35 Å square and a height of 40 Å.

## Discussion

In the present study, we established procedures for the overexpression and purification of *Arabidopsis* MCA proteins using a baculovirus expression system with insect Sf9 cells. The MCA proteins expressed in Sf9 cells retained their activity to mediate Ca^2+^ uptake into cells. We were able to purify MCA2-6H by two-step chromatography in a detergent-solubilized state. The stoichiometry of the MCA2-6H protein in a detergent-solubilized state was confirmed to be a tetramer by combining size-exclusion chromatography, SDS-PAGE under non-reducing conditions, and chemical cross-linking analysis. This result is consistent with our previous finding that non-tagged MCA2 expressed in yeast cells formed a tetramer under the non-reducing conditions of SDS-PAGE [19]. Cryo-EM imaging followed by single-particle reconstruction was then performed to reveal its molecular shape in 3D.

The 3D model of MCA2-6H thus reconstructed is of considerable structural interest. This model depicts two distinct regions with different dimensions: one is a small region and the other a large one. Our recent studies suggest that the small region could be the pore of the channel. A previous study on the structure-function relationship of MCA1 and MCA2 revealed that the N-terminal regions of both proteins, MCA1^1-173^ and MCA2^1-173^, were necessary and sufficient for Ca^2+^ influx when expressed in yeast cells [19]. A unique putative TM segment (named TM1) that is comprised of 23 amino acid residues (Ile^11^ to Ala^33^) exists in this region and may participate in the formation of a pore. The segment contains one acidic amino acid residue, Asp^21^, which may be involved in Ca^2+^ coordination, and the replacement of this residue by Asn results in the complete and partial loss of Ca^2+^ influx activity in MCA1 and MCA2, respectively. In addition, our recent study on membrane topology predicted that TM1 is a unique TM segment in the overall sequence of MCA1 and MCA2 and that the whole region downstream of TM1 is located in the cytoplasm (manuscript in preparation). Taken together, these findings and the present results on the formation of a tetramer (Figure 3) indicate that the small region of the MCA2-6H model is a pore made by four TM1 segments.

The large region of the MCA2-6H model (Figure 6) could be a cytoplasmic regulatory region. As mentioned above, the region downstream of TM1 has been predicted to be in the cytoplasm. This region contains an EF hand-like motif and coiled-coil motif around the middle of the full length of MCA1 and MCA2 and a cysteine-rich region called the Plac8 or DUF614 motif near the carboxyl terminus of the two proteins. The results of our recent study with a yeast assay system suggested that both the EF hand-like motif, which could potentially sense cytoplasmic Ca^2+^ concentrations to self-regulate MCA1 and MCA2, and the coiled-coil motif, which could potentially take part in protein-protein interactions, regulate the Ca^2+^ influx activity of the two proteins, although in different fashions [19]. The role of the plac8 motif is unknown in plants, although the mammalian plac8 protein composed of 112 amino acid residues has been shown to associate with the transcription factor C/EBPβ and bind to the *C/EBPβ* promoter [47].

We attempted to locate the carboxyl terminus of MCA2 using genetically fused EGFP sequence. Expression and purification were performed in exactly the same manner as in MCA2-6H and the protein showed a slightly smaller retention volume in size-exclusion chromatography, which is reasonable for the additional EGFP portion. However, the resulting 3D model did not show the additional volume expected for the EGFP portion (data not shown). This may have been due to the flexibility of the EGFP portion, which was fused via a flexible linker.

The 3D model of MCA2-6H presented had a small pore region. However, a crystal structure, the proton channel protein M2 from influenza A virus, has been reported to have such a small TM region [48]. M2 is a 97 amino acid residue protein with a single TM and the channel is formed by a tetramer. We superimposed the tertiary structure of the M2 tetramer onto our 3D model in Figure 6D. It fit well to the small region of our model.

In summary, we depicted a 3D structural model of MCA2-6H, an MS channel candidate in *Arabidopsis*. This model together with other findings from our recent studies suggests that the tetramer of MCA2 forms a channel with a small TM region and large cytoplasmic region, which contains potential regulatory motifs. Because of similarities in the primary structure and motifs of MCA1 and MCA2, the 3D structure of MCA1 is expected to be similar to that of MCA2. The MCA protein family is found in many species in the plant kingdom and some MCA homologs has been studied in terms of their physiological function (reviewed in [6]). The present study provides some structural and mechanistic insights into mechanosensing and mechanotransduction.
